# Wearable Sensor–Based Gait Analysis in Benign Paroxysmal Positional Vertigo: Quantitative Assessment of Residual Dizziness Using the φ-Bonacci Framework

**DOI:** 10.3390/life16010075

**Published:** 2026-01-04

**Authors:** Beatrice Francavilla, Sara Maurantonio, Nicolò Colistra, Luca Pietrosanti, Davide Balletta, Goran Latif Omer, Arianna Di Stadio, Stefano Di Girolamo, Cristiano Maria Verrelli, Pier Giorgio Giacomini

**Affiliations:** 1 Division of Otolaryngology, University Hospital of Rome Tor Vergata, Viale Oxford 81, 00133 Rome, Italy; saramaurantonio@gmail.com (S.M.); balletta.davide@gmail.com (D.B.); goran.omer@univsul.edu.iq (G.L.O.); sdigirolamo66@gmail.com (S.D.G.); giacominipg@gmail.com (P.G.G.); 2 Department of Electronic Engineering, University of Rome Tor Vergata, Via del Politecnico 1, 00133 Rome, Italy; nicolo.colistra@gmail.com (N.C.); luke.peterst@gmai.com (L.P.); verrelli@ing.uniroma2.it (C.M.V.); 3 Department of Clinical Sciences, College of Medicine, University di Sulaimani, Sulaymaniyah 46001, Iraq; 4 Department Life and Health Science, Link University, 00165 Roma, Italy

**Keywords:** Benign Paroxysmal Positional Vertigo (BPPV), residual dizziness, wearable sensors, gait analysis, φ-bonacci index, vestibular rehabilitation

## Abstract

**Background**: Benign Paroxysmal Positional Vertigo (BPPV) is the most common vestibular disorder. Although canalith repositioning procedures (CRPs) typically resolve positional vertigo, several patients still report imbalance or residual dizziness, which remain difficult to quantify with standard tests. Wearable inertial sensors now allow high-resolution, objective gait analysis and may detect subtle vestibular-related impairments. **Objectives**: This study evaluates the clinical usefulness of sensor-based gait metrics, enhanced by the newly developed φ-bonacci index framework to quantify gait changes and residual dizziness in BPPV before and after CRPs. **Methods**: Fifteen BPPV patients (BPPV-P) and fifteen age-matched controls completed walking tests under eyes-open (EO) and eyes-closed (EC) conditions using wearable inertial measurement units (IMU). φ-bonacci index components—self-similarity (A1), swing symmetry (A2), and double-support consistency (A4)—were calculated to assess gait harmonicity, symmetry and stability. **Results**: Before treatment, BPPV-P exhibited significantly higher A1 values than healthy controls (*p* = 0.038 EO; *p* = 0.011 EC), indicating impaired gait harmonicity. After CRPs, A1 values normalized to control levels, suggesting restored gait self-similarity. Under visual deprivation, both A1 and A4 showed pronounced increases across all groups, reflecting the contribution of vision to balance control. Among post-treatment patients, those reporting residual dizziness demonstrated persistently elevated A4 values—particularly under EC conditions—indicating incomplete sensory reweighting despite clinical recovery. **Conclusions**: Wearable sensor–derived φ-bonacci metrics offer sensitive, objective markers of gait abnormalities and residual dizziness in BPPV, supporting their use as digital biomarkers for diagnosis, rehabilitation, and follow-up.

## 1. Introduction

Benign paroxysmal positional vertigo is the most common vestibular disorder, characterized by brief episodes of vertigo triggered by specific head movements. Although canalith repositioning procedures, such as the Epley maneuver, effectively resolve the acute positional vertigo episodes, subtle yet clinically significant impairments in balance and gait stability frequently persist [1]. These persistent impairments, often described as residual dizziness, involve ongoing sensations of imbalance and instability that can significantly affect patients’ daily activities, quality of life, and risk of falls [2]. Despite their prevalence, residual symptoms remain challenging to detect and objectively quantify by conventional clinical assessments, leading to underestimation and inadequate clinical management [3].

The emergence of wearable inertial measurement units (IMUs) has introduced a promising objective methodology for evaluating gait and balance disturbances in various vestibular and neurological disorders. Wearable sensors precisely capture detailed spatiotemporal gait parameters, detecting subtle alterations that conventional assessments often overlook, thus providing valuable insights into vestibular function and rehabilitation outcomes [4,5,6]. Furthermore, wearable sensors have been successfully employed in neurological research, demonstrating their utility in quantifying gait variability, asymmetry, and instability, and enhancing clinical diagnosis and patient management [7]. A systematic review highlighted their diagnostic accuracy in monitoring gait, balance and fall risk in patients with Parkinson’s disease, stroke, Alzheimer’s disease, and multiple sclerosis, demonstrating their potential as objective tools in both clinical and research settings [8]. Quantitative gait analysis using wearable sensors offers a precise and objective tool for detecting subtle gait impairments in Parkinson’s disease. This approach allows clinicians to distinguish between motor subtypes, track disease progression over time, and identify early gait alterations that conventional assessments may overlook, thereby facilitating personalized interventions and optimizing patient care [9]. The study by Gawrońska et al. introduces an innovative approach to the use of motion sensors, transforming them from simple measurement tools into interactive devices for diagnosis, feedback, and rehabilitation. This perspective is particularly relevant for the management of BPPV and residual dizziness, where dynamic monitoring and personalized feedback can significantly improve postural stability and patients’ quality of life [10].

Recent studies illustrated the potentialities of the φ-bonacci gait index, an innovative gait analysis metric integrating aspects of gait recursivity, self-similarity, symmetry, and consistency through the mathematical properties of generalized Fibonacci sequences and the golden ratio [11,12]. This index has been proved to be sensitive and effective in differentiating gait patterns of BPPV-P from healthy control subjects (HCS) and in monitoring their improvements after CRPs [13]. Despite these promising results, the integration of wearable sensor-based gait analysis into routine vestibular clinical practice remains underexplored.

Building on previous studies that established the feasibility of wearable sensor–based gait analysis in BPPV, the present work extends existing approaches by focusing on the component-level interpretation of φ-bonacci gait indices rather than on global gait descriptors alone. While earlier wearable-sensor studies have documented gait and balance alterations in BPPV, the clinical interpretation of specific gait features associated with residual dizziness remains largely unexplored. In particular, it is still unclear which aspects of gait harmonicity, symmetry, and double-support control are most sensitive to vestibular compensation following canalith repositioning maneuvers. To address this gap, gait was assessed under both eyes-open and eyes-closed conditions to probe sensory reweighting during locomotion, and quantitative gait features were examined in relation to the presence of residual dizziness after treatment. Together, these elements contribute to a more clinically interpretable characterization of gait alterations associated with vestibular compensation.

This study aims to evaluate the clinical usability of wearable sensor–based gait analysis enhanced by the φ-bonacci gait number in patients with BPPV, with the goal of objectively characterizing gait and balance alterations associated with vestibular compensation.

## 2. Materials and Methods

### 2.1. Study Population

Patients with a diagnosis of BPPV and HCS were prospectively recruited from the Department of Otorhinolaryngology, Policlinico Tor Vergata (Rome, Italy). All patients underwent a comprehensive vestibular evaluation including a detailed clinical history and bedside examination consisting of tests for spontaneous, gaze-evoked, and head-shaking nystagmus; positional testing with Dix–Hallpike and supine Roll maneuvers; head impulse test (HIT); and Romberg test.

Inclusion criteria for the BPPV group were: (i) confirmed diagnosis according to the Bárány Society diagnostic criteria, defined by the presence of typical positional vertigo and nystagmus consistent with canalolithiasis on either the Dix–Hallpike or Roll test; (ii) independent ambulation without assistance; and (iii) absence of neurological, otologic (e.g., Ménière’s disease, vestibular neuritis), or systemic comorbidities (e.g., orthopedic or cardiovascular disorders) that could interfere with balance or gait. Exclusion criteria included atypical nystagmus patterns, inability to comply with the gait testing protocol, and use of vestibular suppressant medications within 48 h prior to testing.

CRPs were performed in all BPPV-P by experienced otolaryngologists according to standardized protocols: the Epley maneuver for posterior canal BPPV and the Lempert maneuver for horizontal canal BPPV. HCS were recruited among hospital staff and family members of patients and were included only if they had no vestibular or neurological symptoms in the preceding six months and demonstrated a normal vestibular examination. Follow-up assessment was conducted approximately two weeks after CRPs, including repetition of the complete bedside vestibular examination, positional testing, and HIT to confirm symptom resolution. The final cohort consisted of 15 BPPV-P (mean age, 58.8 ± 5.3 years) and 15 age-matched healthy controls (mean age, 59.4 ± 7.3 years). All participants provided written informed consent. The study was approved by the institutional ethics committee and conducted in accordance with the Declaration of Helsinki.

### 2.2. Experimental Protocol

Participants performed standardized gait assessments along a straight, level hallway at their self-selected comfortable walking speed under two visual conditions: (i) a 20 m walk with EO and (ii) a 10 m walk with EC. The eyes-closed walking condition was included to selectively challenge vestibular and proprioceptive contributions to gait. The protocol was standardized across all participants and performed under supervision to minimize anxiety-related or behavioral confounders.

BPPV-P were evaluated both before and two weeks after CRPs, whereas HCS completed a single session comprising both EO and EC tests under identical conditions. All tests were performed under medical supervision to ensure participant safety and procedural consistency. For BPPV-P, treatment consisted of canalith repositioning maneuvers (Epley for posterior canal and barbecue for horizontal canal involvement) executed by trained otolaryngologists following clinical practice guidelines. Two weeks after the procedure, a follow-up evaluation was performed including vestibular examination and positional testing. To explore potential correlations between subjective residual dizziness and gait indices, post-treatment patients were divided into two subgroups according to their VAS score: VAS = 0 (no dizziness) and VAS > 0 (residual dizziness). The dichotomization of VAS scores (0 vs. >0) was adopted to distinguish asymptomatic patients from those reporting any degree of residual dizziness, in line with the exploratory aim of identifying objective gait correlates of symptom persistence rather than symptom severity.

### 2.3. Data Acquisition

All participants were equipped with wearable inertial sensors from the Movit System G1 motion capture platform (Captiks, Rome, Italy) during the walking assessments to enable quantitative gait analysis. The Movit System G1 integrates multiple synchronized sensing modalities, including triaxial accelerometers, gyroscopes, magnetometers, quaternion processors, and barometric sensors, providing comprehensive kinematic data. Each compact, wireless inertial unit offers 13 degrees of freedom (DOF) and transmits data via a USB-based wireless receiver to the acquisition software [14,15,16].

Motion data were recorded and processed using the Motion Studio and Motion Analyzer software suites (version 2.4), specifically designed for data collection and sensor fusion with this system [17]. The software outputs time-synchronized datasets in .csv format containing sequential timestamps for heel-strike and toe-off events of both limbs, along with angular kinematic data describing the relative orientation of the foot and tibia. These parameters were subsequently used for computation of the φ-bonacci gait index on the composite gait cycle located at the midpoint of each walking trial.

A summary of the sensor specifications, trial characteristics, and analyzed gait parameters is reported in Table 1.

### 2.4. φ-Bonacci Gait Index Components

**Figure 1:**
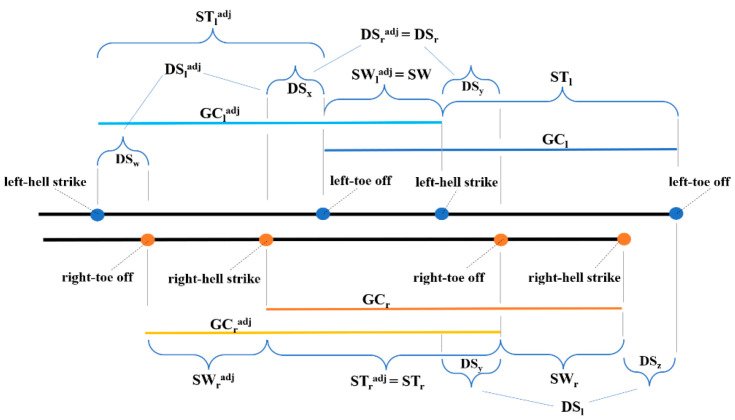
Composite gait cycle: right and left gait cycles and adjoint right and left gait cycles.

Although the full mathematical formulation of the φ-bonacci gait number is reported for methodological completeness, the present study primarily focuses on the physiological interpretation of its internal components. In the context of walking gait as described by previous studies [18,19] the following abbreviations were adopted: GC (gait cycle), HS (heel-strike), TO (toe-off), with subscripts *r* and *l* indicating the right and left sides, respectively. The term adj refers to the adjoint gait cycle, while ST and SW represent the stance and swing phases, while DS indicates the double support phase. Figure 1, adapted from Verrelli et al. [19], schematically illustrates the composite gait cycle, which integrates two overlapping pairs of cycles—right and left (GC*_r_* and GC*_l_*) and their adjoint counterparts (GC^adj^*_r_* and GC^adj^*_l_*). The stance and swing phases for the right and left legs are denoted as ST*_r_*, ST*_l_*, SW*_r_*, and SW*_l_*. The durations of the double support phases for the right (DS*_r_*) and left (DS*_l_*) legs are defined as DS*_r_* = DS*_x_* + DS*_y_* and DS*_l_* = DS*_y_* + DS*_z_*, where DS*_x_*, DS*_y_*, and DS*_z_* are illustrated in Figure 1. The assumption of equal partitioning of the double support sub-phases (DS*_x_* = DS*_y_*, and consequently DS*_y_* = DS*_z_*) reflects the principle of double support consistency. Similarly, the durations of the adjoint right and left gait cycles—ST^adj^*_r_*, ST^adj^*_l_*, SW^adj^*_r_*, SW^adj^*_l_*, DS^adj^*_r_*, and DS^adj^*_l_*—are also represented in Figure 1.

Verrelli et al. [19] introduced the φ-bonacci gait number (Y_φ_), a novel mathematical descriptor for the composite gait cycle, rooted in the generalized finite-length Fibonacci sequences and the golden ratio (φ). The full expression of this index, denoted as Y_φ_ and detailed in Equation (10) of Verrelli [19], emerges from a new experimental framework involving an extended fractal analysis of gait, which takes into account the spatial relationship between the foot and the tibia. This formulation represents the most natural extension of the gait ratio |SW/DS − φ| introduced in [18,20] for symmetric gait patterns, broadening its application to asymmetric and non-recursive walking dynamics. Additionally, it integrates a weighted correction similar to the index |ΔSW|/SW proposed by [21], applied consistently to both the gait cycle and its adjoint. Table 2 reports its complete version, where λ, δ, μ^adj^, λ^adj^, and ν^conj^ are positive weights (Such weights play the role of gains and can be freely chosen by the user, in accordance with the specific analysis requirements: in this study, they are all set to 1, in line with the original formulation proposed by [11], to ensure an equal and balanced contribution of each component within the comprehensive φ-bonacci index), and the normalized quantity is given by:

(1) $${\left(\frac{X_{n}}{X_{d}}-X_{v}\right)}_{n}^{2}={\left(\frac{X_{n}}{X_{d}}\right)}^{-1}{\left(\frac{X_{n}}{X_{d}}-X_{v}\right)}^{2}$$

in terms of positive reals X*_n_*, X*_d_*, X*_v_* (where *n* generically stands for numerator, *d* stands for denominator, and v stands for value), whereas the positive real numbers z_1_, z_2_, z_3_ denote the time distances from the corresponding left or right heel-strikes and toe-off instants of the three time instants representing the minima of angular positions (with negative signs) of the left and right feet relative to the tibias (with a 90 degrees-angle between foot and tibia being plotted at 0-degrees). Table 2 shows Y_φ_ as a weighted combination of four distinct components: (i) the first term captures the influence of self-similarity in gait generation; (ii) the second reflects the contribution of swing phase symmetry; (iii) the third represents an enhanced self-similarity effect; and (iv) the fourth quantifies double support consistency—specifically, the symmetry between the two double support sub-phases: (i) left foot trails and right foot leads; (ii) right foot trails and the left foot leads.

Values of the full or simplified φ-bonacci indices that are close to zero indicate varying degrees of gait recursiveness, harmonicity (self-similarity), swing symmetry, and double-support balance, depending on which terms are included in the computation. Generally, lower values are associated with more harmonic, symmetric, and consistent walking patterns, while higher values are typically observed in abnormal or pathological gaits.

In this study, for the first time, we examine the internal components of the composite φ-bonacci index related to self-similarity (referred to as A1), swing symmetry (referred to as A2), and double support consistency (referred to as A4). Table 2 also reports the mathematical definitions of A1, A2, and A4 in their normalized forms.

**Remark** **1.** *According to [19], Y_φ_ draws inspiration from the experimental findings reported in [22], which indicate that physiological symmetric walking is characterized by a stance phase duration approximating 62% of the gait cycle, a swing phase lasting about 38%, and a double support phase constituting approximately 24% of the gait cycle. Furthermore, the occurrence of a minimum angular position (negative in value) of the foot relative to the tibia (where a 90-degree angle between the foot and tibia is defined as 0 degrees) happens at around 7% of the gait cycle duration within each double support sub-phase, with the complementary interval lasting roughly 5%. Interestingly, this structure aligns with the Fibonacci sequence (with the fixed point φ) as evidenced in the sequence:* $5\;\times 2=10\;\left(\frac{1}{\varphi^{5}}\approx 9.018\right);\;7\;\times 2=14\;\left(\frac{1}{\varphi^{4}}\approx 14.591\right);\;24\;\left(\frac{1}{\varphi^{3}}\approx 23.608\right);\;38\;\left(\frac{1}{\varphi^{2}}\approx 38.198\right);\;62\left(\frac{1}{\varphi }\approx 61.804\right);\;100.$

In this study, the contribution of A3 was not considered, as the analysis was specifically focused on the components related to swing symmetry (A2), double-support consistency (A4), and gait harmonicity (A1) in quantifying residual dizziness. Indeed A3 reflects the angular positioning of the foot relative to the tibia, a feature occurring within each double-support subphase, whose investigation was beyond the aims of the present work.

### 2.5. Signal Processing and Statistical Analysis

Temporal gait parameters, obtained from the Motion Analyzer software (version 2.4) through the processing and fusion of raw sensor signals, were imported into a custom algorithm developed in MATLAB (The MathWorks, Natick, MA, USA, version R2023b). This algorithm detects, for each subject, the precise time points of HS and TO events for both the left and right foot, corresponding to the gait cycle and its adjoint phases on each side. These HS and TO timestamps were then used to compute the φ-bonacci gait number, including its components A1, A2, and A4 for all composite gait cycles identified during the entire walking trial for each participant.

To reduce the influence of potential measurement noise and transient gait fluctuations, the analysis focused on the composite gait cycle located at the midpoint of each subject’s walking sequence. Statistical analysis and data visualization were carried out using GraphPad Prism Software Version 9. The results, reported in the following sections, refer to the index and contribution values computed on these central gait cycles. Since the data did not meet normality assumptions (assessed using the Kolmogorov–Smirnov test), a non-parametric Mann–Whitney U test was applied to evaluate differences between the mean values of each pair of sample groups. Statistical significance was defined by a *p*-value threshold of less than 0.05. Furthermore, to assess the diagnostic capability of the indices and contributions in discriminating HCS from pathological ones, Receiver Operating Characteristic (ROC) curve analyses were performed using the Wilson/Brown method. Index performance was quantified using the area under the ROC curve (AUC), with values greater than 0.5 considered statistically meaningful. The optimal threshold for the ROC curve was determined using the Youden index, with a likelihood ratio (LR = Sensitivity/(1 − Specificity)) greater than 2 used as the criterion. The chosen threshold c maximized the value of Sensitivity + Specificity − 1.

## 3. Results

A total of 15 patients with BPPV and 15 HCS completed the walking assessment protocol. All participants tolerated the wearable sensor examination without adverse events. CRPs were effective in all patients, and post-treatment clinical reassessment confirmed complete resolution of positional nystagmus.

### 3.1. Groups Comparison

**Figure 2:**
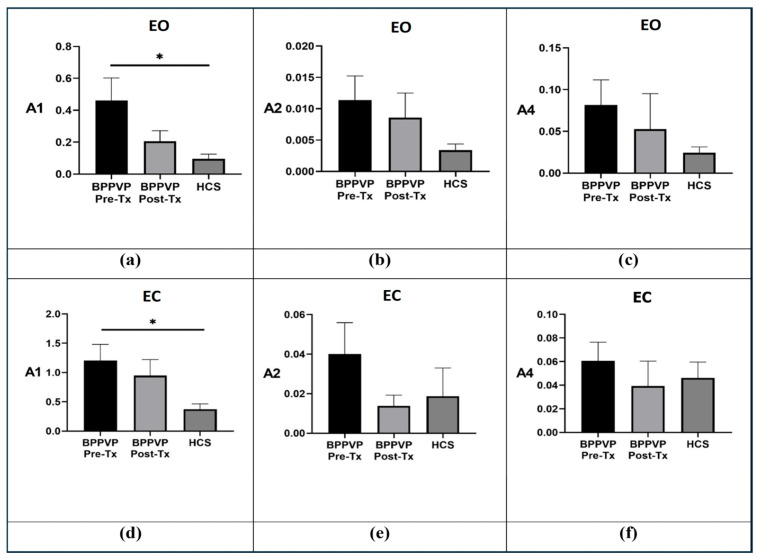
Mean ± SEM of the A1, A2, and A4 values computed for BPPV-P pre-tx (black), BPPV-P post-tx (light grey), and HCS (dark grey). Panels (**a**–**c**) refer to the EO condition, whereas panels (**d**–**f**) refer to EC condition. Comparison between HCS (dark grey), BPPV-P pre-tx (black), and BPPV-P post-tx (light grey) in EO and EC conditions for index A1, A2, and A4. Statistically significant differences are highlighted by means of asterisks (* = *p*-value < 0.05).

Before comparing groups, we first assessed whether the φ-bonacci index components remain stable across adjacent gait cycles. As shown in Table 3 and Table 4, the values of A1, A2, and A4 computed on the central, preceding, and subsequent gait cycles exhibit minimal variability in both BPPV-P pre-tx, BPPV-P post-tx, and HCS under EO. This confirms the robustness of the central gait cycle as a reliable representation of steady-state walking gait cycle, providing a solid methodological basis for the group comparisons that follow. Figure 2 summarizes the comparison among BPPV-P before treatment (BPPV-P pre-tx), after treatment (BPPV-P post-tx), and HCS under both EO and EC conditions for the A1, A2, and A4. In the EO condition, all indices exhibited consistent differences across groups; however, a statistically significant difference was found only for the A1 index between BPPV-P pre-tx and HCS (*p* = 0.038). Under the EC condition, a similar pattern was observed, with a significant difference for A1 between BPPV-P pre-tx and HCS (*p* = 0.011). No significant differences were observed between BPPV-P post-tx and HCS in either condition, suggesting a recovery of healthy gait patterns following CRPs.

### 3.2. Diagnostic Performance Analysis

**Figure 3:**
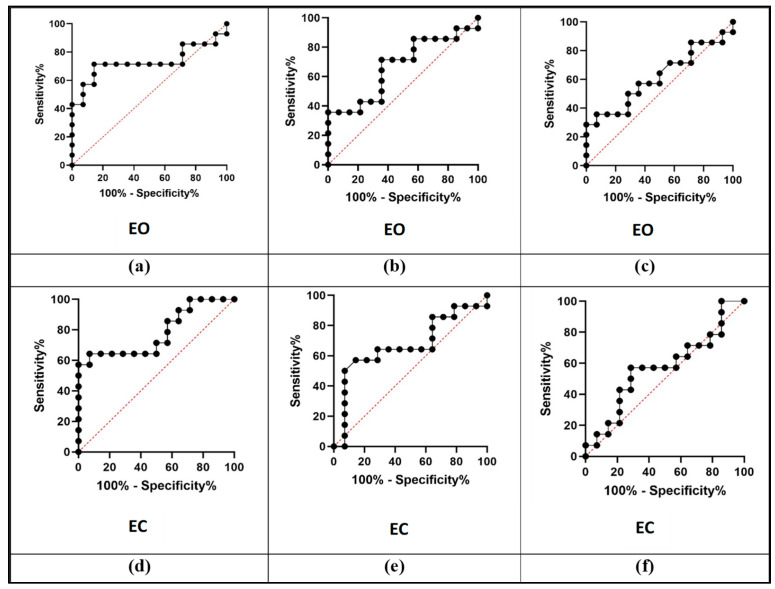
ROC curves used to test classification performance of indices A1, A2, and A4 for BPPV-P pre-tx vs. HCS. Panels (**a**–**c**) refer to EO condition, whereas panels (**d**–**f**) refer to EC condition. ROC curves used to test classification performances of indices A1, A2, and A4 for BPPV-P pre-tx vs. HCS in EO and EC conditions.

Receiver operating characteristic (ROC) curve analysis (Figure 3) demonstrated variable classification performances across indices. The A1 index showed the highest discriminative ability in distinguishing BPPV-P pre-tx from HCS, with an area under the curve (AUC) of 0.749 under EO (Specificity = 71.43%; 95% CI Specificity = 45.35% to 88.28%; Sensitivity = 85.71%; 95% CI Sensitivity = 60.06% to 97.46%; Threshold = 0.2070) and 0.780 under EC (Sensitivity = 64.29%; 95% CI Sensitivity = 38.76% to 83.66%; Specificity = 92.86%; 95% CI Specificity = 68.53% to 99.63%; Threshold = 0.8650). The A2 and A4 indices exhibited lower but still positive classification trends, suggesting partial sensitivity to gait alterations associated with vestibular dysfunction.

### 3.3. Effect of Visual Deprivation

**Figure 4:**
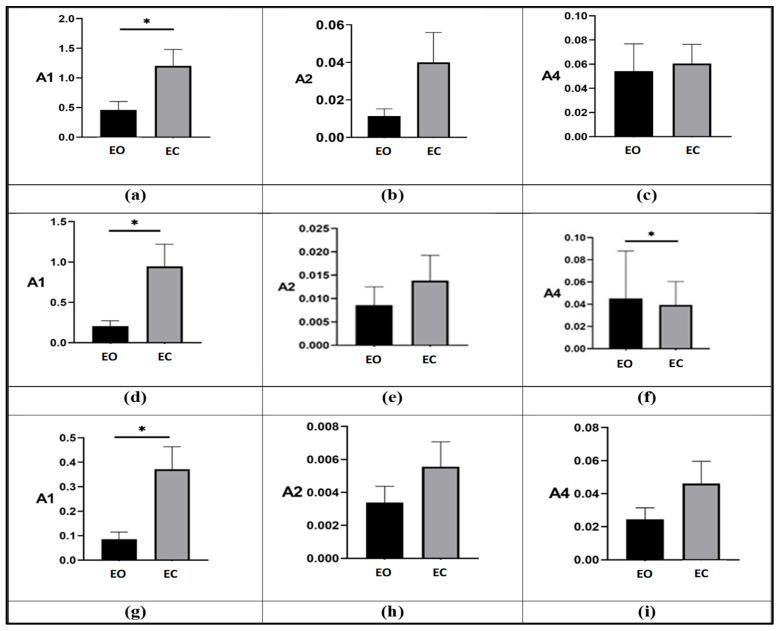
Comparison between EO test (black) and EC test (grey) for the three indices A1, A2, and A4 for BPPV-P pre-tx (**a**–**c**), BPPV-P post-tx (**d**–**f**), and HCS (**g**–**i**). Statistically significant differences are highlighted by means of asterisks (* = *p*-value < 0.05).

Figure 4 compares performance between EO and EC tests within each group. Across all participants, index values increased when walking with EC, reflecting diminished gait performance in the absence of visual feedback. Table 5 reports the mean percentage increase between EO and EC for each index and group. The most pronounced variation was observed for A1, with mean percentage increases of 419% in BPPV-P pre-tx, 1400% in BPPV-P post-tx, and 470% in HCS. The A2 index showed minimal change in HCS (2.1%) but increased markedly in BPPV-P pre-tx (213%) and post-tx (489%). The A4 index increased across all groups, with the greatest change in BPPV-P post-tx (144%).

### 3.4. Residual Dizziness Analysis

Residual dizziness was reported by a subset of patients after treatment despite clinical resolution of positional vertigo, according to their VAS score. Given the exploratory nature of this analysis, results are presented descriptively. Median values for A1, A2, and A4 under both EO and EC conditions are presented in Table 6. Except for A1 in the EO condition, all indices exhibited lower values in patients without residual dizziness, with the largest intergroup difference observed for A4 under EC (−90.6%). These findings highlight the indices’ ability in detecting and quantifying gait differences even among clinically recovered patients.

## 4. Discussion

The use of wearable inertial sensors has substantially advanced quantitative gait analysis, enabling objective and high-resolution assessment of locomotor performance in clinical settings. In vestibular disorders, these technologies offer a unique opportunity to capture subtle impairments in balance control and sensory integration that are frequently undetectable using conventional bedside examination or static posturography. Consistently with the paradigm of precision vestibular medicine, sensor-derived gait parameters are increasingly regarded as digital biomarkers capable of tracking functional impairment and recovery [23,24,25,26] as well as enhanced postural control and fall prevention through the use of wearable force sensors and biofeedback systems [27,28]. The reliability and reproducibility of inertial sensors in quantifying postural sway further support their superiority over static posturography for the evaluation of dynamic balance [29]. Despite these advances, earlier wearable-sensor studies in BPPV have largely focused on static balance or basic spatiotemporal gait descriptors, providing limited insight into the mechanisms underlying dynamic gait organization and post-treatment recovery. Within this technological framework, the φ-bonacci index represents an innovative analytical approach for gait quantification. Grounded in the mathematical principle of self-similarity, it integrates temporally harmonic components of walking into physiologically interpretable indices that reflect self-similarity and symmetry of movement. Previous studies have demonstrated its sensitivity to neuromotor coordination and postural control alterations, suggesting potential applications in vestibular and neurological diseases. In the study by Mohammed et al., wearable sensors were used to monitor patients’ balance and movements in real time, providing objective data to assess residual instability after BPPV episodes and to guide personalized rehabilitation programs [30]. In the present study, φ-bonacci–index components were able to distinguish pre-treatment BPPV patients from healthy control subjects and to capture gait normalization following canalith repositioning. Within this context, the present study extends previous work by adopting a component-level analysis of the φ-bonacci framework, focusing on self-similarity (A1), swing symmetry (A2), and double-support consistency (A4) during walking under both eyes-open and eyes-closed conditions. This approach enables a more direct assessment of sensory reweighting during locomotion and provides clinically interpretable information on gait organization. Among the evaluated parameters, the A1 index—reflecting self-similarity—was the most responsive to disease state. Its elevation in the pre-treatment phase and subsequent normalization after therapy indicate that A1 captures transient disruption of gait automaticity due to vestibular imbalance and its recovery after central adaptation. These findings reinforce the concept that wearable-based indices can serve as sensitive functional markers of dynamic vestibular compensation. From a methodological perspective, although the cohort size was necessarily limited by the clinical timing of BPPV diagnosis, the φ-bonacci index components nonetheless demonstrated statistically meaningful discriminative performance (AUC 0.75–0.78), supporting the adequacy of the sample for this exploratory ROC-based analysis. The consistency of effect sizes across groups further indicates that even a modest sample can capture physiologically relevant differences, including those related to residual dizziness. Although pre- and post-treatment observations were obtained from the same subjects, a single representative gait cycle was extracted for each condition, resulting in independent summary measurements rather than repeated correlated observations. For this reason, a non-parametric Mann–Whitney U test was applied, which is appropriate for independent distributions in small-sample exploratory studies.

The A4 index, representing double-support consistency, increased across all conditions when visual feedback was removed, consistent with a stabilization strategy adopted to maintain balance under sensory constraint. The magnitude of this increase was greater in BPPV-P, even after successful repositioning, suggesting persistent dependence on visual or proprioceptive cues during locomotion. This pattern parallels the clinical observation that some patients remain visually dependent or experience subtle disequilibrium despite full symptomatic remission. Importantly, these results confirm that wearable-derived parameters can reveal residual abnormalities that are not captured by conventional posturographic or clinical measures.

The comparison between EO and EC walking provided additional insights into the mechanisms of sensory integration. In all groups, the transition to visual deprivation led to higher φ-bonacci values, reflecting the central role of vision in stabilizing gait. However, the disproportionate increase observed in BPPV-P indicates incomplete recalibration of vestibular input and an overreliance on visual control. The larger variability of A1 and A4 under EC conditions likely reflects impaired sensory reweighting—a process that normally enables efficient balance control when one sensory modality is reduced or unavailable. Although visual deprivation may induce cautious gait, the uniform application of the eyes-closed condition across groups and the disproportionate response observed in symptomatic patients support its interpretation as a probe of sensory reweighting rather than a non-specific behavioral effect.

Despite the efficacy of canalith repositioning procedures in resolving positional vertigo, a considerable subset of patients with BPPV continues to experience residual dizziness, typically described as mild imbalance, unsteadiness, or motion-induced discomfort. This condition has been widely reported in the literature, with an estimated prevalence of 30–60% following successful repositioning maneuvers [31]. Its underlying mechanisms are still debated but are thought to involve delayed central adaptation of vestibular input, transient mismatch among visual, proprioceptive, and vestibular cues, or temporary dysfunction of the velocity storage mechanism [32]. Although these symptoms are usually self-limited, resolving within two to four weeks, they represent a meaningful clinical phenomenon that can impair quality of life and delay functional recovery. In this cohort, all patients who reported residual dizziness after canalith repositioning exhibited complete clinical recovery, with absence of positional nystagmus on follow-up examination. Nevertheless, their φ-bonacci indices—particularly A4 during EC walking—remained elevated compared with those of asymptomatic patients. This finding supports the hypothesis that residual dizziness does not reflect persistent otoconial debris but rather a transient inefficiency in multisensory integration, primarily involving the reweighting of vestibular, proprioceptive, and visual inputs. Similar mechanisms have been proposed in the early stages of persistent postural-perceptual dizziness (PPPD), a functional vestibular disorder characterized by sustained non-spinning vertigo and visual-motion sensitivity due to maladaptive sensory reweighting [33]. In patients with other vestibular disorders such as Ménière’s disease, abnormal pupil dilation during walking has been observed, indicating increased cognitive effort required to maintain balance with EC. This phenomenon, related to postural instability, suggests that motion sensors could be useful for objectively assessing and monitoring balance difficulties even in BPPV-P post-tx [34]. Although the short duration of symptoms in the present study does not fulfill PPPD diagnostic criteria, the observed alterations in A4 under visual deprivation may represent a temporary, reversible analog of these maladaptive processes. Clinically, this finding supports the rationale for targeted vestibular rehabilitation protocols focused on reducing visual dependence, such as balance training under altered sensory conditions, dynamic gait exercises with progressive visual constraint, and multisensory integration tasks. In recent times, vestibular research has increasingly shifted from predominantly subjective clinical assessments toward quantitative and predictive models based on wearable sensor data. Accelerometers, inertial measurement units, and wearable force or pressure sensors allow the acquisition of rich kinematic and kinetic information that can be integrated into machine-learning pipelines for classification, monitoring, and prediction of gait and balance impairments [35,36]. Typical workflows include signal preprocessing, extraction of relevant features—such as step duration, cadence, symmetry indices, acceleration variability, harmonic ratios, and stability measures—and subsequent training of supervised models [35,37]. In BPPV specifically, recent studies have shown that accelerometer-based gait features combined with machine-learning approaches can quantify walking instability and classify symptom-related gait alterations, with performance influenced by sensor placement and task conditions [35,37]. Moreover, wearable plantar force sensors combined with machine-learning techniques have been used to characterize compensatory postural strategies and enhance predictive performance for postural control assessment and rehabilitation monitoring [36,38,39]. Within this evolving framework, φ-bonacci–index gait components may be viewed as higher-order, physiologically interpretable features describing gait organization, rhythmicity, and stabilization strategies, and represent promising features for future machine-learning applications. From a clinical standpoint, the φ-bonacci framework demonstrates significant translational potential. The indices are easily derived from a short, wearable-based walking test and provide reproducible, physiologically meaningful outputs. The normalization of A1 following therapy supports its role as a biomarker of recovery, while elevated A4 values may identify patients at risk for residual imbalance who could benefit from targeted rehabilitation to enhance vestibular weighting and reduce visual dependence. Integration of these indices into clinical workflow could complement standard vestibular assessment by providing objective endpoints for treatment efficacy and long-term follow-up.

### Limitations and Future Directions

The study has several limitations. The sample size was relatively small and restricted to a single-center cohort, limiting generalizability. This limited number of participants reflects the clinical constraints of recruiting BPPV patients at the time of confirmed diagnosis; although small, the cohort still provided statistically meaningful differences, but future multi-center studies with larger populations are needed to strengthen generalizability. These findings are intended to serve as a foundation for future hypothesis-driven studies and larger validation cohorts. Although the φ-bonacci index yields interpretable parameters, normative datasets stratified by age and sex are not yet established. Future research should validate these findings in larger and more diverse populations, evaluate longitudinal trends during rehabilitation, and determine the minimal clinically important differences for each index.

Given their sensitivity to gait harmonicity, symmetry, and double-support stability, the φ-bonacci metrics represent promising features for future machine-learning applications. These indices could support classification of pathological vs. recovered gait patterns, prediction of residual dizziness or imbalance, and the development of personalized rehabilitation strategies.

The present findings should be interpreted within an exploratory and mechanistic framework. Rather than proposing a ready-to-use clinical tool, this study aims to identify which specific components of gait organization are most sensitive to vestibular imbalance and early post-treatment sensory reweighting in BPPV. In this context, the differential behavior of the self-similarity (A1) and double-support consistency (A4) components provides insight into distinct aspects of dynamic gait control that may be differentially affected during the acute and recovery phases of the disorder. Incorporating these quantitative measures into otoneurological practice could refine the evaluation of balance function, support individualized rehabilitation strategies, and advance the development of data-driven models for recovery in vestibular medicine.

## 5. Conclusions

This study demonstrates that wearable sensor–based gait analysis enhanced by the φ-bonacci framework offers a sensitive and objective method to quantify gait and balance alterations in patients with BPPV. Also, according to Zhang et al., wearable technology offers a promising approach to assess gait disturbances in BPPV-P in clinical settings [35]. The φ-bonacci gait index components, particularly the self-similarity component (A1) and the double-support consistency (A4), effectively differentiated pre-treatment patients from HCS and captured gait normalization following the canalith repositioning maneuver. Under visual deprivation, both A1 and A4 exhibited marked percentage increases, with the effect being particularly pronounced for A4 in patients reporting residual dizziness.

Importantly, these results should be regarded as exploratory and hypothesis-generating. While not sufficient to support immediate clinical implementation, they identify specific gait components that warrant further investigation as potential functional markers of vestibular imbalance and recovery. Further validation in larger cohorts is warranted to establish normative values and confirm their broader applicability across vestibular and neurological disorders.

## Figures and Tables

**Table 1:** Summary of dataset characteristics, sensor specifications, and gait analysis parameters.

Category	Parameter	Description
Participants	Number of subjects	15 BPPV-P, 15 HCS
	Age (mean ± SD)	BPPV-P: 58.8 ± 5.3 yr; HCS: 59.4 ± 7.3 yr
Sensor system	Platform	Movit System G1 (Captiks, Guidonia Montecelio, Italy)
	Sensor modalities	3D accelerometer, gyroscope, magnetometer, barometric sensor, quaternion fusion processor
	Degrees of freedom	13 DOF per IMU
	Sampling rate	200 Hz
	Wireless communication	USB-based wireless receiver
Walking trials	EO condition	20 m walk at self-selected comfortable speed
	EC condition	10 m walk at self-selected comfortable speed
	Trial duration	Distance covered at comfortable walking pace
Data output	Extracted parameters	HS/TO timestamps (L/R),
	Export format	CSV synchronized data
Gait cycle analysis	Number of gait cycles analyzed	1 composite gait cycle at midpoint of each trial

***Note*****.** The midpoint gait cycle was selected because it represents the portion of the walking trial farthest from start- and end-related transients, where both IMU-related measurement errors and gait transient effects are minimal. This central gait cycle therefore provides the most stable and representative segment for computing the φ-bonacci gait index and its internal components A1, A2, and A4.

**Table 2:** Mathematical expression for the φ-bonacci gait number.

$\Upsilon_{\varphi }=\sqrt{{\left(\frac{SW_{l}}{DS_{r}}-\varphi \right)}_{n}^{2}+{\left(\frac{SW_{r}}{DS_{l}}-\varphi \right)}_{n}^{2}+{\mu^{adj}\left(\frac{SW_{r}^{adj}}{DS_{l}^{adj}}-\varphi \right)}_{n}^{2}}+\lambda \sqrt{{\left(\frac{SW_{r}}{SW_{l}}-1\right)}_{n}^{2}+\lambda^{adj}{\left(\frac{SW_{r}^{adj}}{SW_{r}}-1\right)}_{n}^{2}}$ $+\nu^{conj}\sqrt{{\left(\frac{DS_{r}}{z_{1}+z_{2}}-\varphi \right)}_{n}^{2}+{\left(\frac{DS_{l}}{z_{1}+z_{2}}-\varphi \right)}_{n}^{2}}+\delta \sqrt{{\left(\frac{DS_{x}}{DS_{y}}-1\right)}^{2}}$
$A_{1}={\left(\frac{SW_{l}}{DS_{r}}-\varphi \right)}^{2}\cdot {\left(\frac{SW_{l}}{DS_{r}}\right)}^{-1}+{\left(\frac{SW_{r}}{DS_{l}}-\varphi \right)}^{2}\cdot {\left(\frac{SW_{r}}{DS_{l}}\right)}^{-1}+{\left(\frac{SW_{r}^{adj}}{DS_{l}^{adj}}-\varphi \right)}^{2}\cdot {\left(\frac{SW_{r}^{adj}}{DS_{l}^{adj}}\right)}^{-1}$
$A_{2}={\left(\frac{SW_{r}}{SW_{l}}-1\right)}^{2}\cdot {\left(\frac{SW_{r}}{SW_{l}}\right)}^{-1}+{\left(\frac{SW_{r}^{adj}}{SW_{r}}-1\right)}^{2}\cdot {\left(\frac{SW_{r}^{adj}}{SW_{r}}\right)}^{-1}$
$A_{3}={\left(\frac{DS_{r}}{z_{1}+z_{2}}-\varphi \right)}^{2}\cdot {\left(\frac{DS_{r}}{z_{1}+z_{2}}\right)}^{-1}+{\left(\frac{DS_{l}}{z_{1}+z_{2}}-\varphi \right)}^{2}\cdot {\left(\frac{DS_{l}}{z_{1}+z_{2}}\right)}^{-1}$
$A_{4}={\left(\frac{DS_{x}}{DS_{y}}-1\right)}^{2}\cdot {\left(\frac{DS_{x}}{DS_{y}}\right)}^{-1}$

**Table 3:** φ-bonacci index components (A1, A2, A4) computed on the preceding, central, and subsequent gait cycles for BPPV-P (n = 15, each row) in pre- and post-tx under EO.

EO BPPV-P pre-tx								
A1			A2			A4		
0.635	0.629	0.647	0.00102	0.00100	0.00104	0.00390	0.00400	0.00404
0.022	0.022	0.021	0.00006	0.00007	0.00007	0.00888	0.00900	0.00899
0.023	0.023	0.024	0.00335	0.00330	0.00341	0.00096	0.00010	0.00009
0.235	0.230	0.223	0.01051	0.01060	0.01021	0.07495	0.07160	0.06855
0.932	0.920	0.901	0.00346	0.00350	0.00341	0.03529	0.03660	0.03749
0.006	0.007	0.006	0.00057	0.00056	0.00054	0.01090	0.01090	0.01132
0.702	0.714	0.721	0.03175	0.03160	0.03312	0.21320	0.20580	0.20796
0.499	0.479	0.485	0.00664	0.00650	0.00671	0.03817	0.03890	0.03735
0.527	0.532	0.522	0.00267	0.00270	0.00279	0.02473	0.02590	0.02540
0.005	0.005	0.004	0.00140	0.00140	0.00143	0.11641	0.12200	0.12378
0.321	0.329	0.325	0.00271	0.00273	0.00266	0.00060	0.00060	0.00060
0.304	0.309	0.309	0.03596	0.03690	0.03867	0.31073	0.31540	0.30169
2.021	2.013	1.998	0.04020	0.04030	0.04200	0.00255	0.00250	0.00245
0.237	0.231	0.229	0.01898	0.01830	0.01801	0.30355	0.30100	0.30219
0.459	0.463	0.456	0.01140	0.01145	0.01170	0.08160	0.08190	0.08070
EO BPPV-P post-tx								
A1			A2			A4		
0.655	0.654	0.652	0.00008	0.00008	0.00008	0.00040	0.00040	0.00040
0.015	0.015	0.015	0.00087	0.00090	0.00086	0.00611	0.00600	0.00606
0.002	0.002	0.002	0.00328	0.00330	0.00316	0.00191	0.00190	0.00189
0.157	0.157	0.158	0.01864	0.01860	0.01859	0.08691	0.08450	0.08591
0.385	0.386	0.389	0.04357	0.04240	0.04351	0.59356	0.60000	0.61369
0.012	0.012	0.015	0.00061	0.00064	0.00063	0.0	0.0	0.0
0.002	0.002	0.001	0.00296	0.00300	0.00309	0.01540	0.01500	0.01546
0.151	0.149	0.151	0.00067	0.00069	0.00068	0.00101	0.00100	0.00101
0.016	0.016	0.015	0.00108	0.00105	0.00107	0.00170	0.00170	0.00184
0.011	0.012	0.011	0.00009	0.00009	0.00009	0.01177	0.01180	0.01119
0.470	0.471	0.480	0.00048	0.00047	0.00045	0.00010	0.00010	0.00010
0.065	0.067	0.067	0.00065	0.00065	0.00067	0.00767	0.00740	0.00759
0.701	0.683	0.678	0.04018	0.04030	0.04028	0.00069	0.00070	0.00068
0.252	0.254	0.256	0.00759	0.00776	0.00783	0.00685	0.00700	0.00690
0.206	0.205	0.206	0.00900	0.00895	0.00900	0.05350	0.05380	0.05410

**Table 4:** φ-bonacci index components (A1, A2, A4) computed on the preceding, central, and following gait cycles for HCS (n = 15, each row) under EO.

EO HCS								
A1			A2			A4		
0.181	0.183	0.182	0.00900	0.00920	0.00940	0.04990	0.05090	0.05190
0.052	0.053	0.052	0.00084	0.00086	0.00088	0.00045	0.00047	0.00048
0.006	0.006	0.007	0.00013	0.00013	0.00014	0.05980	0.06060	0.06110
0.388	0.390	0.395	0.00069	0.00071	0.00072	0.00136	0.00140	0.00143
0.065	0.067	0.068	0.00595	0.0061	0.00625	0.06040	0.06170	0.06300
0.008	0.009	0.008	0.00940	0.00940	0.00960	0.07170	0.07390	0.07370
0.007	0.007	0.007	0.00189	0.00190	0.00194	0.0088	0.00900	0.00920
0.049	0.05	0.049	0.00009	0.00010	0.00010	0.0077	0.00790	0.00810
0.187	0.184	0.182	0.00068	0.00069	0.00071	0.0110	0.01130	0.01150
0.060	0.058	0.056	0.00367	0.0037	0.0037	0.00740	0.00760	0.00780
0.045	0.043	0.042	0.00226	0.0023	0.00235	0.0318	0.03220	0.03260
0.027	0.027	0.028	0.00970	0.0099	0.01010	0.0197	0.02010	0.02050
0.015	0.016	0.016	0.00077	0.00078	0.00079	0.00223	0.00230	0.00236
0.242	0.242	0.245	0.00166	0.00170	0.00174	0.00176	0.00180	0.00183
0.117	0.118	0.120	0.00307	0.00314	0.00320	0.02790	0.02831	0.02873

**Table 5:** Mean percentage differences between EO test and EC test for all indices across all groups.

**Group**	ΔA1	ΔA2	ΔA4
BBPVP pre-tx	419%	213%	48.6%
BBPVP post-tx	1400%	489%	144%
HCS	470%	2.1%	30%

**Table 6:** Median values of A1, A2, and A4 for subjects with and without residual dizziness after treatment in both EO and EC conditions, and percentage differences between the two groups.

	A1		A2		A4	
	**EO**	**EC**	**EO**	**EC**	**EO**	**EC**
**No Residuals**	0.082	0.205	0.000795	0.0058	0.00145	0.00185
**Residual dizziness**	0.067	0.526	0.00105	0.007991	0.0017	0.0197
**Δ** **%**	+22.38806	−61.0266	−24.2857	−27.4183	−14.7059	−90.6091

## Data Availability

The data that support the findings of this study are available on request.

## Glossary

The following abbreviations are used in this manuscript:

BPPV	Benign Paroxysmal Positional Vertigo
BPPV-P	Patients with Benign Paroxysmal Positional Vertigo
HCS	Healthy Control Subjects
CRP	Canalith Repositioning Procedure
HIT	Head Impulse Test
VAS	Visual Analogue Scale
IMU	Inertial Measurement Unit
DOF	Degrees of Freedom
EO	Eyes Open
EC	Eyes Closed
GC	Gait Cycle
HS	Heel Strike
TO	Toe Off
ST	Stance Phase
SW	Swing Phase
DS	Double Support Phase
adj	Adjoint Gait Cycle
Yφ	φ-Bonacci Gait Number
A1	Self-Similarity Component
A2	Swing Symmetry Component
A4	Double-Support Consistency Component
X_n_, X_d_, X_v_	Positive real quantities representing numerator (n), denominator (d), and value (v), used in the normalized computation of the φ-bonacci index
z1, z2, z3	Time distances between the angular minima of the foot–tibia trajectory and the corresponding heel-strike or toe-off events
λ, δ, μ^adj^, λ^adj^, ν^conj^	Weighting coefficients of the φ-bonacci index
